# Supplementary material for: Crystallization of polarons through charge and spin ordering transitions in 1T-TaS2
Source: Nat Commun. 2023 Nov 3;14:7055. doi: 10.1038/s41467-023-42631-6 (PMC10624925; doi:10.1038/s41467-023-42631-6)
Supplement: Supplementary file 1 — Supplementary Information [file 41467_2023_42631_MOESM1_ESM.pdf]

Supplementary Information

Crystallization of polarons through charge and spin ordering transitions in 1T-TaS_2

E.S. Bozin[1], M. Abeykoon[2], S. Conradson[3], G. Baldinozzi[4], P. Sutar[3] and D. Mihailovic[3]

[1] *Condensed Matter Physics and Materials Science Division, Brookhaven National Laboratory, Upton, NY 11973, USA*
[2] *Photon Sciences Division, Brookhaven National Laboratory, Upton, NY 11973, USA*
[3] *Dept. of Complex Matter, Jozef Stefan Institute, Jamova 39, SI-1000 Ljubljana, Slovenia*
[4] *Centralesupélec, CNRS, SPMS, Université Paris-Saclay, bât Eiffel, Gif-sur-Yvette, Île-de-France, 91190, FRANCE*

1. SUPPLEMENTARY FIGURES AND TABLES 2

Supplementary Figure 1. Polymorphic transformations in 1T-TaS2 on warming in the M phase. 6

Supplementary Figure 2. Evolution of stacking correlations in 1T-TaS2. 3

Origin of c-axis negative thermal expansion (NTE) jumps 4

Supplementary Figure 3. Possible origin of the c-axis NTE jumps. 4

Supplementary Figure 4. Comparison of PDF data of 1T-TaS_2 in the low temperature regime. 5

Supplementary Figure 5. Local structure changes across M-IC transition in 1T-TaS2. 6

Supplementary Table 1. Details of the single layer 13×13 supercell $P3$ model. 7

Supplementary Figure 6. Features of the $P3$ structure fit to the 15 K PDF data of 1T-TaS_2 in the C state. 8

Supplementary Figure 7. Considerations of the fitting range and the applicability of the $P3$ approximant model. 9

2. ORDER PARAMETERS DESCRIBING THE SYMMETRY CHANGE LEADING TO THE COMMENSURATE CDW LOW TEMPERATURE PHASE OF 1T-TAS_2. 11

Revisiting the 1T (single layer) C-CDW: atomic vibrations 11

The low T variants 12

Symmetry adapted coordinates of Ta displacements at q_1 and at q_1+q_2. 15

Cartesian components of the displacements of each independent Ta atom in the daughter phase 16

Supplementary table 2. Fractional displacement contributions originating from the amplitudes transported at q_1 and q_1+q_2. 17

1. Supplementary Figures and Tables

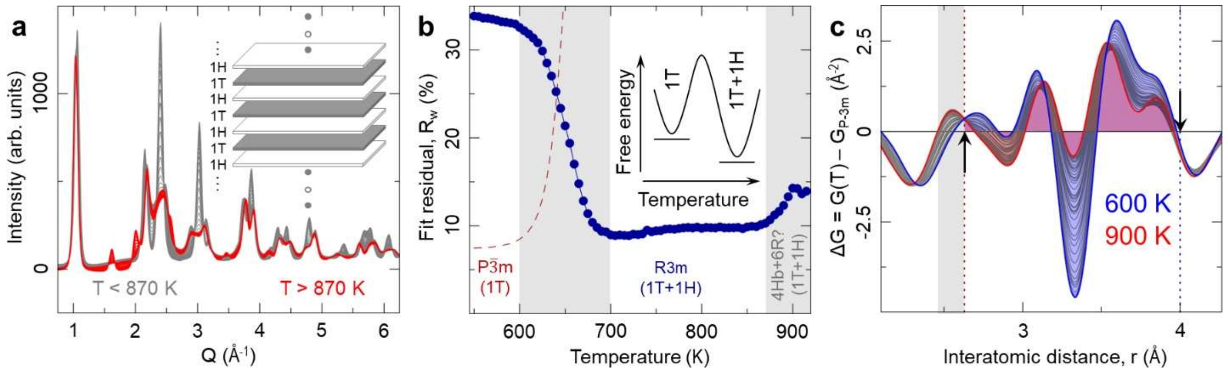

Supplementary Figure 1. Polymorphic transformations in 1T-TaS_2 on warming in the M phase.

a Stack of diffraction patterns revealing two polymorphic transformations on warming, T > 600 K. Red stack data, T > 870 K, reveal additional transformation to that focused on in the main text. This additional restructuring likely involves a distorted mixture of 4Hb (P63/mmc) and 6R (R3m) polymorphs with significant stacking faults. Low resolution of our diffraction data did not allow unambiguous characterization. Tentative PDF assessment suggested a complex mixture of alternating 1T and 1H layers (inset). **b** Transformation temperatures charted by fit residuals of $P\overline{3}m$ and R3m models, as indicated in the panel. Inset: sketch of the free energy of TaS_2, depicting metastable state (1T) and more stable configurations (1T+1H) achieved as temperature is elevated. **c** Stack of narrow *r*-range difference PDFs, ΔG, obtained by subtracting simulated PDF from a literature reference based on undistorted $P\overline{3}m$ at 300 K, from experimental PDFs (lower r-resolution, Q_{max}=20 $Å^{-1}$) collected at T > 600 K. The difference PDF is acquired for all M-phase data. To estimate the change in fraction across the polymorphic transformations, the difference PDF, ΔG, was integrated between 2.6 – 4 Å (isosbestic points encompassing PDF feature of interest, between vertical arrows) for each temperature, and the final integral was normalized to the value obtained at 600 K. The uncertainties in the distorted fraction were estimated by extending the integration range over the grey window (nearest low-*r* zero crossings) and repeating the process. A double of the maximal value of such uncertainty was adopted as the error bars shown in the inset of Fig. 2b of the main text.

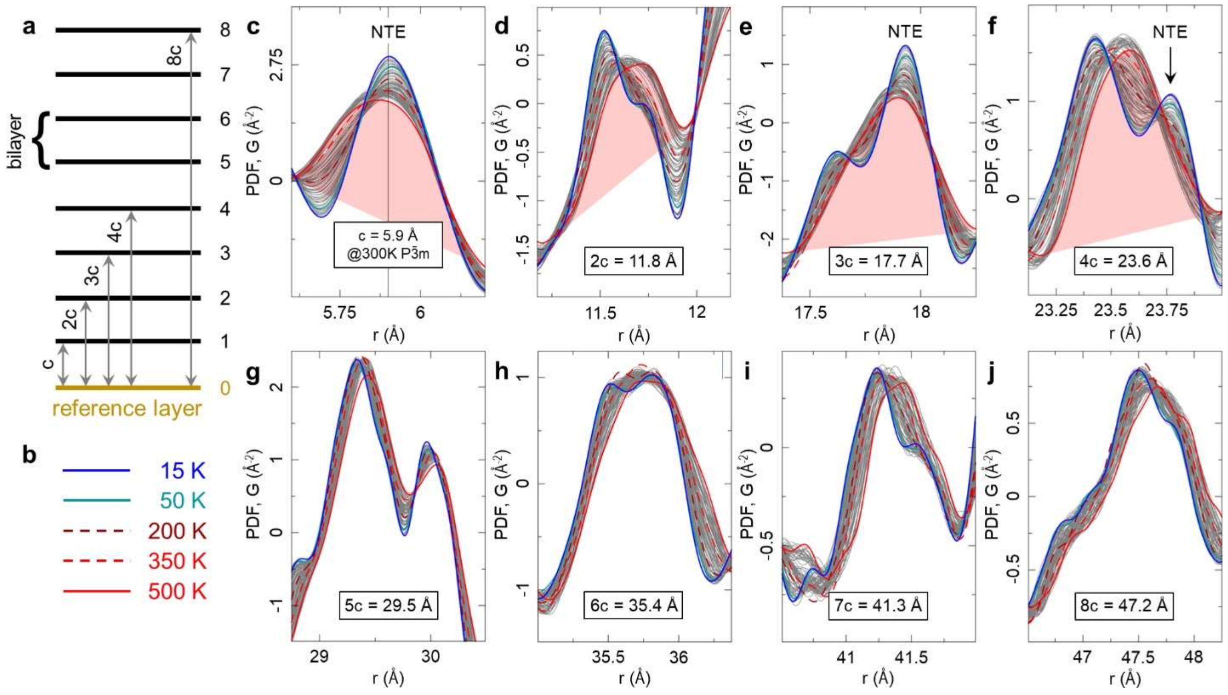

Supplementary Figure 2. Evolution of stacking correlations in 1T-TaS_2.

a Idealized stacking of Ta-layers, with interlayer spacings as indicated, assigned based on $P\bar{3}m$ structure, and defined with respect to a reference layer. **b** Legend of color-coded temperatures of selected *r*-range PDF data shown in panels **c**-**j**. In these panels the r-ranges were selected to encompass features associated with the multiples of the $P\bar{3}m$ c-axis lattice parameter reported in literature for 300 K. While the PDF traces corresponding to the transitions to equilibrium electronic phases are colour coded, the other data in the stacks are shown in grey. The correlations up to 8c are shown. Profiles at 500 K are highlighted in red up to 4c range to emphasize the broadness of the high temperature signal which in part originates from interlayer disorder. Features with apparent negative thermal expansion characteristics are labelled as NTE.

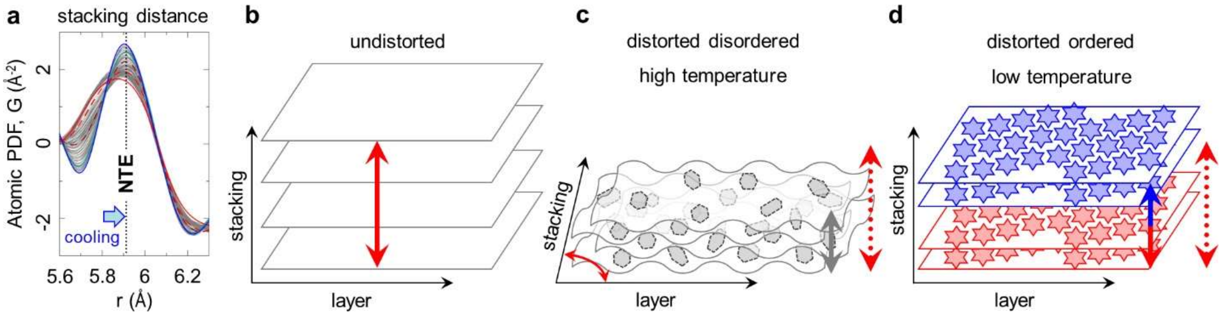

Supplementary Figure 3. Possible origin of the c-axis NTE jumps.

a Stack of PDF data around the nearest neighbour layer stacking distance at ~5.9 Å, as presented in Fig. 4a of the Main Text, and in Suppl. Fig. 3c. Dashed vertical line marks c-axis lattice parameter in undistorted P$\bar{3}$m structure. Coloured traces mark 500 K (solid red line), 350 K (dashed red line), 200 K (dashed dark red line), 50 K (solid cyan line) and 15 K (solid blue line). **b.** In P$\bar{3}$m structure layers are equidistant, as sketched. **c.** In distorted heavily disordered structure, such as that observed at higher temperatures, puckering distortions result in heavily distorted layers, resulting in interlayer stacking disorder and observed broad distribution of nearest neighbour interlayer distances, such as seen in **a.** In proposed NTE model this results in layer-shears and monoclinic compressions yielding shorter-than-expected average c-lattice parameter. **d.** As interlayer orders set in on cooling, the puff-pastry-like stacking disorder gets lifted, and the c-axis expands resulting in observed NTE jumps, as described in text.

Origin of c-axis negative thermal expansion (NTE) jumps

Dilatometry measurements in 1T-TaS2 [1] revealed anomalous NTE jumps in c-axis parameter, most significantly at the NC-C transition, but also at the IC-NC transition, origin of which has not been understood to date. Our data also reveal NTE behavior of certain PDF features, as marked in Figs. 2b,d,e and 4a of the Main Text, as well as in Supplementary Figure 3, and offer a simple explanation for this phenomenon. Based on these observations we propose a disorder-based rationale for NTE jumps. In putative undistorted P$\bar{3}$m crystal structure 1T layers stacked along c-axis are equidistant, Suppl. Fig. 4b. However, as our PDF analysis demonstrates, 1T-TaS2 is locally distorted at all temperatures at which this metastable polymorph exists, including the highest temperature in the M phase, resulting in 1T*-TaS2. There, the distortions involve local puckering of Ta layers, such as schematically shown the top panel of Fig. 4f in the Main Text as well as in Supp. Fig. 4c. As a consequence, disordered layers exhibit a range of interlayer distances and concomitant sheer displacements due to Coulomb repulsion of polarons in neighbouring layers, evident from broad interlayer distance distributions that are, importantly, centered at nominal distance shorter than simple integer multiples of undistorted P$\bar{3}$m c-axis lattice parameter (Main Text Fig. 4a,c at high temperature, and also Supplementary Fig. 3c and 4a) resulting in, on average, closer packing of the layers. Due to this, the experimentally measured reference c-axis lattice parameter at high temperature is anomalously short, resembling puff-pastry configuration as depicted in Fig. 4 f (top) and Supp. Fig. 4c. As polaron gas condenses into a

Wigner crystal, and the distortions become long range ordered and stacked, aided by Coulomb repulsion effects, the ordered layers space away, effectively removing monoclinic shears, resulting in increase of the average interlayer spacing (Main Text Fig. 4 a,c at low temperature), as sketched in Supp. Fig. 4d, leading to apparent NTE jumps observed in dilatometry.

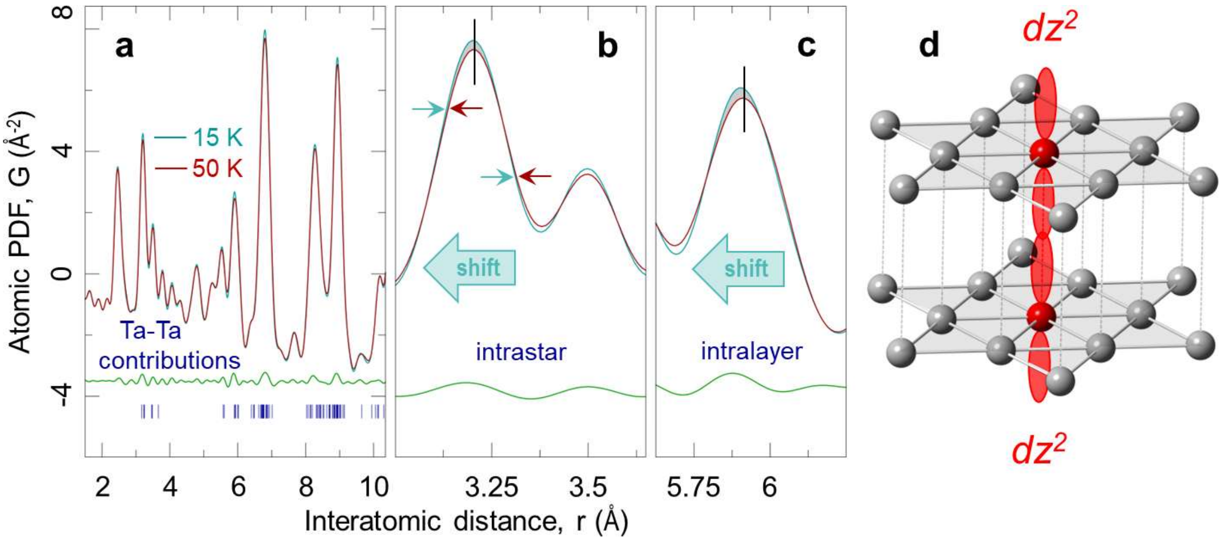

Supplementary Figure 4. Comparison of PDF data of 1T-TaS_2 in the low temperature regime.

a An overplot of experimental PDFs of 15 K (cyan trace) and 50 K (red trace), with corresponding differential (green trace) offset for clarity. The Ta-Ta contributions per $P\overline{3}m$ model used in this work are shown as blue ticks. **b** The *r*-region sensitive to intrastar correlations. Apart from sharpening, expected upon temperature reduction, centroid of the relevant peak also shifts to the left, consistent with SoD regularization revealed by modelling show in the Main Text. **c** The *r*-region sensitive to the nearest neighbour intra-bilayer SoD correlations. The peak also exhibits observable shifting, consistent with enhanced inter-bilayer binding. Vertical black line in **b** and **c** marks the centroid of feature of interest at 50 K. One possible origin of this behaviour, as discussed in the Main Text, is a formation of spin-singlet dimers associated with the stray spins, presumably from the dz^2 manifolds, in two adjacent SoDs within the bilayers. Notably, the amount of change in PDF signal seen in **c** is quite large. It is unlikely than only one Ta-Ta contact per SoD (1:13) contracts, implying that the mechanism is more complex than simple pairing of spins in dz^2 orbitals portrayed in **d**. Importantly, the features in the differential in **a** are well structured, stronger under some PDF peaks and weaker under others. Given that the differential is of two data sets, it is unlikely that they originate from artifacts such as termination ripples or noise in the data. Even if present, such effects would effectively cancel out in the subtraction, leaving only signature of subtle structural differences. We further note that as part of the experiment we carried out a counting statistics test to assess the signal to noise ratio. In this, data statistics was found to be satisfactory after counting for 10 seconds. The actual counting time was 120 seconds, an order of magnitude longer.

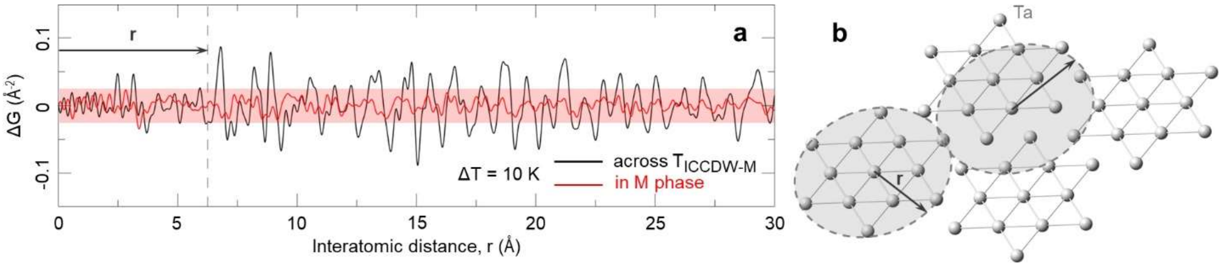

Supplementary Figure 5. Local structure changes across M-IC transition in 1T-TaS_2.

a Comparison of difference PDF between data that are 10 K apart within metallic phase (T > 550 K) and across the IC-M transition, implicating existence of local distortions in the M phase over at least a radius r, as sketched in **b**. Note that these difficult-to-model distortions do not reflect star or David in the M phase, but heavily puckered hexagonal discs as indicated by modelling presented in the Main Text. Enhancement of difference signal on the length-scale r > 5 Å implies growth of structural correlations in the IC regime.

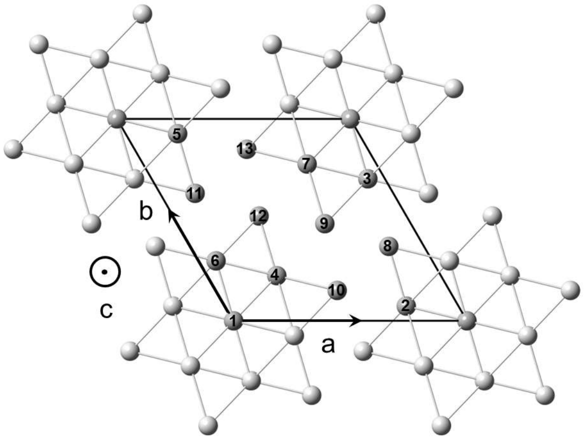

Tantalum	x	y	z
1	-	-	-
2	p1	p2	p3
3	1-p2	p1-p2	p3
4	1-p1+p2	1-p1	p3
5	1-p1	1-p2	-p3
6	p2	1-p1+p2	-p3
7	p1-p2	p1	-p3
8	p4	p5	p6
9	1-p5	p4-p5	p6
10	1-p4+p5	1-p4	p6
11	1-p4	1-p5	-p6
12	p5	1-p4+p5	-p6
13	p4-p5	p4	-p6

Sulfur	x	y	z	Sulfur	x	y	z
14	p7	p8	p9	27	1-p14	p13-p14	p15
15	1-p8	1+p7-p8	p9	28	1-p13+p14	1-p13	p15
16	-p7+p8	1-p7	p9	29	1-p13	1-p14	1-p15
17	1-p7	1-p8	1-p9	30	p14	1-p13+p14	1-p15
18	p8	-p7+p8	1-p9	31	p13-p14	p13	1-p15
19	1+p7-p8	p7	1-p9	32	p16	p17	p18
20	p10	p11	p12	33	1-p17	1+p16-p17	p18
21	1-p10	p10-p11	p12	34	-p16+p17	1-p16	p18
22	1-p10+p11	1-p10	p12	35	1-p16	1-p17	1-p18
23	1-p10	1-p11	1-p12	36	p17	-p16+p17	1-p18
24	p11	1-p10+p11	1-p12	37	1+p16-p17	p16	1-p18
25	p10-p11	p10	1-p12	38	-	-	p19
26	p13	p14	p15	39	-	-	1-p19

Supplementary Table 1. Details of the single layer $\sqrt{13} \times \sqrt{13}$ supercell P$\bar{3}$ model.

The sketch of the Ta plane, top left, expanded to show four SoDs, with Ta atoms in the model enumerated. Fractional coordinates of Ta atoms are described by 6 independent positional parameters, with symmetry constrains as tabulated in top right. Fractional coordinates of S atoms are described by 13 independent positional parameters, that were symmetry constrained as tabulated (bottom). The model does not account for the c-axis stacking correlations beyond the nearest neighbour layers and does not explain PDF data beyond the length-scale of ~1 nm. Since the fitting range is shorter than the unit cell size, lattice parameters (a=b, c) provide the distance metrics rather than describing translational symmetry. The parameters are labelled as p_i, where i=1-19 represent the index of each symmetry-independent parameter.

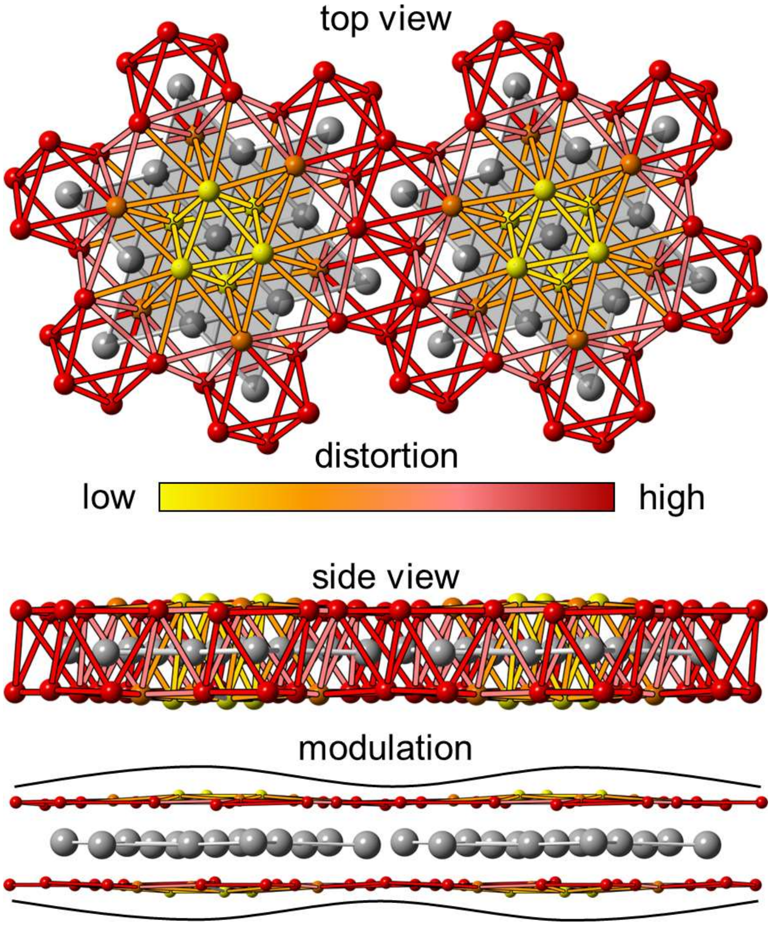

Supplementary Figure 6. Features of the $P\overline{3}$ structure fit to the 15 K PDF data of 1T-TaS_2 in the C state.

The illustration focuses on two SoD motifs. Tantalum atoms are shown as larger gray spheres, whereas sulphur atoms are depicted as smaller coloured spheres, where colouring reflects their location with respect to the central Ta in SoDs. Moving away from the central Ta the TaS_6 octahedra are progressively more distorted, as portrayed by the colour bar which could be considered as coarse indicator of strain associated with the SoD formation. Although our local model is relatively simple approximant that does not describe the long-range ordering, it does incorporate the degrees of freedom necessary to account for the modulation of sulphur sublattice, as shown in the bottom most panel where the sulphur atoms are represented by very small spheres to emphasize the effect seen in the fit. The solid lines are the guides to the eye and the modulation they depict is out of proportion. Such modulation is expected in the long range ordered structure and is enabled in our approximant model focused on the evolution of local Ta-Ta correlations.

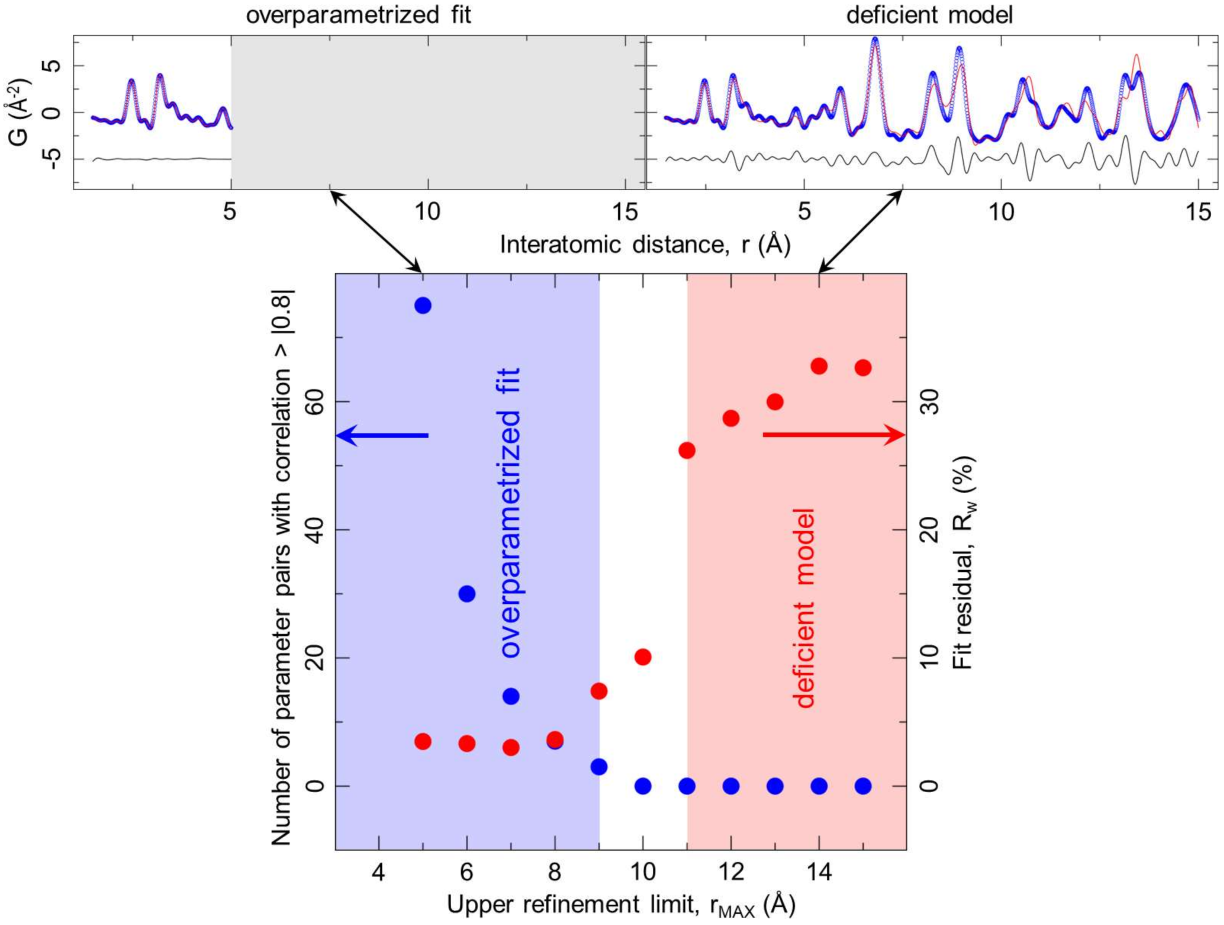

Supplementary Figure 7. Considerations of the fitting range and the applicability of the P$\overline{3}$ approximant model.

In order to establish suitable range for the local structure study, the model was tested against the 15 K dataset. In this, 19 structural parameters, described in Suppl. Fig. 6, were varied, while the lattice parameters, thermal parameters, and the overall scale were kept fixed. The fitting test was conducted over a variable range of data by incrementing the upper limit of refinement, r_{MAX}, by 1 Å, from 5 Å to 15 Å, as shown in the figure. In this process we monitored two quantities: the fit quality captured by the fit residual, R_W, and the number of parameter-pairs whose correlation is larger than |0.8| as reported by the fitting program. In the former, large value of RW indicates inadequacy of the model used. In the latter, parameters exhibiting correlation >|0.8| are typically considered to be unreliable since they are not independent when too narrow a data range is considered. Their report by the fitting platform indicates an over-parametrization of the model or, conversely, insufficient information in the data to constrain the model. In our test, fits of exceptionally good quality were obtained for $r_{MAX} \leq 8$ Å, as shown by the RW(T) dependence (red symbols in the bottom panel, right ordinate) and illustrated in the top left panel. However, fits over these ranges result in heavily over-parametrized fits, as indicated by the temperature dependence of the number of correlated parameter pairs (blue symbols in the bottom panel, left ordinate). Increasing the fitting range results in parameters becoming uncorrelated, as sufficient amount of data is added to the fitting. While this initially

helps, with a moderate jump in R_W, for $r_{MAX} \geq 11$ Å the fits begin to deteriorate, as illustrated in the upper right panel. The reason behind this is that wider range of PDF data provides access to significant contributions of the NNN interlayer correlations which are beyond the scope of our present model. We identified $r_{MAX} = 10$ Å as a sweet spot offering a reasonable compromise between the over-parametrization (model too complex for the data range) and underfitting (model inadequacy). The result is a reliable determination of the local structure, which is the focus of the present work.

2. ORDER PARAMETERS DESCRIBING THE SYMMETRY CHANGE LEADING TO THE COMMENSURATE CDW LOW TEMPERATURE PHASE OF 1T-TaS_2.

The analysis of the symmetry of the high temperature structure of 1T-TaS_2 can help understanding the structural changes of the low temperature daughter structures in terms of the symmetry adapted coordinates belonging to the irreducible representations of the parent space-group symmetry. The physical order parameters describing the change (in this case, the atomic displacements) can be classified as basis functions of the irreducible representations of the parent symmetry group. In the current case, multiple order parameters contribute to the final observed distortion: the individual degrees of freedom provided by an instance of these multi-dimensional order parameters are physically meaningful because they provide a natural description of the distortions of the parent structure.

Lowering the temperature, some of the symmetry elements of the symmetry group of the parent phase are lost (e.g the mirror planes), but the surviving symmetries that describe the daughter phase are a subgroup of the parent phase that is uniquely identified by its combination with the new superlattice basis (and eventual new supercell origin choice). Using a frozen-phonon picture, the structural parameters describing the low temperature daughter structures can be organized as a superposition of polarization vectors containing the cartesian components of the displacements of each one of the independent atoms of the daughter phase.

Revisiting the 1T (single layer) C-CDW: atomic vibrations

In the following, we totally neglect any c-stack rule and consider the symmetry changes occurring in a single layer of the TaS_2 structure. We start from the standard description of the prototype phase that has space group symmetry P-3m1 (#164, D^3_{3d}, $a_P=b_P\approx3.34$ Å, $c_P\approx5.894$ Å, $\gamma=120°$). In this structure, there are two independent atoms with positions described by the coordinates summarized in the table below:

atom	site	x	y	z	sym
Ta0	1a	0	0	0	D3d
S0	2d	1/3	2/3	0.22074	C3v

The mechanical representation Γ at the Brillouin zone centre of this prototype structure is reducible and it consists of 2 acoustic and 4 optic mode frequencies as shown by the decomposition into irreducible representations.

$$\Gamma = (A_{2u} \oplus E_u) \oplus (A_{1g} \oplus A_{2u} \oplus E_g \oplus E_u)$$

In particular, the acoustic phonons of this prototype structure belong to the $(A_{2u} \oplus E_u)$ irreducible representations and involve the displacements of the Ta atoms. The A_{2u} irrep describes a vibration of the Ta atom along *z*, while the E_u symmetry adapted displacements describe degenerate vibrations in the *xy* plane.

The low T variants

All the low temperature phases have axes rotated when compared to the original frame of the high temperature prototype structure. The lattice changes produced by rotation around the z-axis (which has A_{2g} representation) can maintain the -3 symmetry operations, but they systematically remove the mirrors and 2-fold axes of the parent group. The low temperature phases also involve superlattice reflections and therefore require the definition of a superlattice cell in real space. The superlattice reflections characteristic of the low temperature variants (IC, NC, and C-CDW phases) involve a condensation of Bragg intensity at some *q*-points in the high temperature Brillouin zone.

In the simplest description of the low temperature variants (incommensurate or commensurate), two additional independent vectors of the high temperature Brillouin zone are required. The low temperature phase (C-CDW) is obtained by the following matrix transformation of the prototype lattice

$$(a \quad b \quad c) = (a_P \quad b_P \quad c_P) \begin{pmatrix} 4 & -1 & 0 \\ 1 & 3 & 0 \\ 0 & 0 & 1 \end{pmatrix}$$

In this reference cell, there are 13 replicas of the TaS_2 motif. The atomic positions of the prototype structure can be described in this new metric that has symmetry P-3 (#147, a=b≈12.04254 Å, c≈5.894 Å, γ=120°) by a new set of independent atomic positions summarized in the table below:

atom	site	x	y	z
Ta1	1a	0.	0.	0.
Ta2	6g	10/13	1/13	0.00000
Ta3	6g	11/13	5/13	0.00000
S1	6g	-1/3+6/13	1/3-2/13	0.22074
S2	6g	2/3+3/13	1/3-1/13	0.22074
S3	6g	2/3+4/13	1/3+3/13	0.22074
S4	6g	-1/3+7/13	1/3+2/13	0.22074
S5	2d	2/3	1/3	0.22074

This phase corresponds to the lock-in of the components of two vectors q_1 and q_2; these reciprocal space vectors have generic components (ab0) and form an angle of 60° to maintain the symmetry of the threefold axis. Their components have the form q_1= (a, b, 0) and q_2=(C_{-3+} q_1)= (-b, a+b,0). At the lock-in, described by the lattice change transformation matrix, $4q_1$-q_2=a* and q_1+$3q_2$=b* so that q_1=3/13 a*+1/13 b* and q_2=-1/13 a*+4/13 b*.

Since there is no component normal to the layer, the q_1 vector defines a star of 6 vectors (including q_2) related by the symmetry operations of the parent space. The same considerations actually apply also to the symmetry analysis of the incommensurate variant studied by Spijkerman et al. In that case, the components a and b of the vectors are incommensurate but not far from their lock-in values:

a_{IC}=0.2448(2) -> a_{C-CDW}=0.2308=3/13

b_{IC}=0.0681(2) -> b_{C-CDW}=0.0769=1/13

As we can see, at the lock-in, the a_{IC} component has decreased and b_{IC} has increased, so the q_1 and q_2 vectors orientations relative to the parent phase have rotated while the temperature was lowered. A

commensurate approximant of the incommensurate phase with P-3 symmetry could be constructed eventually taking a=1/4=0.25 and b=1/16=0.0625, or some other convenient rational approximation of those values.

To calculate the frozen phonons displacements in the C-CDW phase, we have to consider two stars of vectors. The first star is the one already discussed, originating from q_1 (q_2 obviously belongs to this star by construction) and represented by blue arrows in Supplementary Figure 9. The second star of vectors is the one originating from q_1+q_2 with components (2/13 5/13 0) that indexes the 2nd order superlattice reflections of this phase, so that all the 12 extra spots of the reciprocal lattice of the C-CDW phase can be indexed. Each one of these two stars has in principle 12 branches and so the dimension of the Order Parameter for each one of the two stars. The 12 branches of the star of these vectors in P-3m1 have components E(a,b,0), i(-a, -b, 0), -3^+(-b,a+b,0), 3^+(b, -a-b, 0), -3^- (a+b,-a,0), 3^-(-a-b, a, 0), m_{100}(-a,a+b,0), 2_{100}(a, -a-b, 0), m_{010}(a+b,-b,0), 2_{010}(-a-b, b, 0), 2_{110}(b,a,0), and m_{110}(-b, -a, 0).

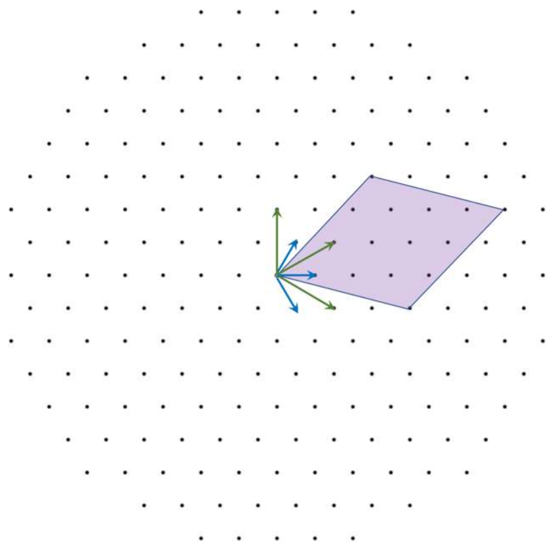

Supplementary Figure 9. Representation of the two stars indexing the superlattice reflections of the C-CDW phase. Elements of the q_1 star are displayed as blue vectors, elements of the q_1+q_2 star are shown as green vectors. The shaded area is the reciprocal cell of the prototype phase.

For the q_1 star we can simply take the spots indexed by the elements (E, -3^+ and -3^-) with explicit components (3/13 1/13 0) (-1/13 4/13 0) (4/13 -3/13 0): therefore, the OP describing the superpositions of the atomic displacements has components (a0a0a0000000). The same choice can be made for the q_1+q_2 star, or explicitly (2/13 5/13 0) (-5/13 7/13 0) (7/13 -2/13 0). The OP describing the transformation from the parent phase to the C-CDW lock-in phase has overall symmetry components

(a0a0a0000000;b0b0b0000000). It is then possible to decompose the atomic displacements of the Ta atoms at any temperature in terms of the amplitudes of the E_u and A_{2u} modes transported at q_1 and at q_1+q_2.

Symmetry adapted coordinates of Ta displacements at q_1 and at q_1+q_2.

These displacements of the Ta atoms are particularly relevant for the description of the dimerisation of Ta distances that is a characteristic signature of the C-CDW phase.

Eu (q_1) 1st component – phases and relative displacement amplitudes along x,y for Ta2 and Ta3 atoms

Ta1 0.0000 0.0000 0.0000

Ta2 -0.9708 -1.0000 0.0000

Ta3 -0.2939 -0.3028 0.0000

Eu (q_1) 2nd component – phases and relative displacement amplitudes along x,y for Ta2 and Ta3 atoms

Ta1 0.0000 0.0000 0.0000

Ta2 -0.5222 0.4778 0.0000

Ta3 -0.1581 0.1447 0.0000

Eu (q_1+q_2) 1st component – phases and relative displacement amplitudes along x,y for Ta2 and Ta3 atoms

Ta1 0.0000 0.0000 0.0000

Ta2 -0.0088 0.2939 0.0000

Ta3 0.0292 -0.9708 0.0000

Eu (q_1+q_2) 2nd component – phases and relative displacement amplitudes along x,y for Ta2 and Ta3 atoms

Ta1 0.0000 0.0000 0.0000

Ta2 -0.3028 -0.1581 0.0000

Ta3 1.0000 0.5222 0.0000

A2u (q_1) – phases and relative displacement amplitudes along z for Ta2 and Ta3 atoms

Ta1 0.0000 0.0000 0.0000

Ta2 0.0000 0.0000 0.3028

Ta3 0.0000 0.0000 1.0000

A2u (q_1+q_2) – phases and relative displacement amplitudes along z for Ta2 and Ta3 atoms

Ta1 0.0000 0.0000 0.0000

Ta2 0.0000 0.0000 -1.0000

Ta3 0.0000 0.0000 0.3028

This analysis can be also extended to S atoms, but:

- more components are involved, reducing the value of the decomposition procedure
- optic phonons do not seem particularly relevant for this phase transition mechanism

Cartesian components of the displacements of each independent Ta atom in the daughter phase

The supplementary table 2 summarizes the fractional displacement contributions originating from the amplitudes transported at q_1 and q_1+q_2. These displacements add to the undistorted positions of the Ta2 and Ta3 atoms inherited from the high temperature parent phase. The displacements of the Ta1 atom at the origin of the lattice are fixed by the symmetry element of the daughter phase, so they are not reported in the table. Some trends can be remarked:

- The out-of-plane z component decreases with decreasing T, resulting in puckering of the Ta atoms
- The Eu q_1 component is more important for Ta2 atoms whereas the Eu (q_1+q_2) component is the dominant displacement component for Ta3 atoms.

	undistorted		q1	175K	150K	125K	100K	50K	15k
Ta2_x	0.07692	Eu	Ta2_dx	-0.00957	-0.00957	-0.00898	-0.00903	-0.00838	-0.00779
Ta2_y	0.30769	Eu	Ta2_dy	-0.01577	-0.01576	-0.01536	-0.01541	-0.01548	-0.01529
Ta2_z	0	A2u	Ta2_dz	-0.00219	-0.00210	-0.00151	-0.00162	-0.00162	-0.00169
Ta3_x	0.15385	Eu	Ta3_dx	-0.00188	-0.00188	-0.00193	-0.00193	-0.00215	-0.00227
Ta3_y	0.61538	Eu	Ta3_dy	0.00291	0.00290	0.00272	0.00273	0.00254	0.00236
Ta3_z	0	A2u	Ta3_dz	-0.00727	-0.00695	-0.00498	-0.00535	-0.00536	-0.00560

	q1+q2	175K	150K	125K	100K	50K	15K
Eu	Ta2_dx	0.00311	0.00320	0.00341	0.00359	0.00392	0.00410

Eu	Ta2_dy	0.00405	0.00404	0.00443	0.00445	0.00446	0.00446
A2u	Ta2_dz	0.00894	0.00877	0.00797	0.00755	0.00722	0.00731
Eu	Ta3_dx	-0.00313	-0.00278	-0.00337	-0.00285	-0.00178	-0.00118
Eu	Ta3_dy	0.01027	0.01056	0.01125	0.01185	0.01295	0.01355
A2u	Ta3_dz	-0.00271	-0.00265	-0.00241	-0.00229	-0.00218	-0.00221

Supplementary table 2. Fractional displacement contributions originating from the amplitudes transported at q_1 and q_1+q_2.

[1] O. Sezerman, A. M. Simpson, and M. H. Jericho, Solid State Commun 36, 737 (1980).
